# TEACHING IN THE TUNDRA: LESSONS LEARNED AT THE UNIVERSITY OF ALASKA ANCHORAGE

**DOI:** 10.1093/geroni/igad104.0638

**Published:** 2023-12-21

**Authors:** Britteny Howell, Rosellen Rosich, Mikki Easley

**Affiliations:** University of Alaska Anchorage, Anchorage, Alaska, United States; University of Alaska Anchorage, Anchorage, Alaska, United States; University of Alaska Anchorage, Anchorage, Alaska, United States

## Abstract

UAA is an open-enrollment, public university of about 12,000 students. The gerontology minor was formed in 2005 as a result of the Geriatric Education Center (GEC). However, the loss of the GEC and most gerontology faculty, coupled with low student enrollment in the minor, led to its deactivation in 2014. Since then, several new faculty have been hired with an interest in gerontology. The Gerontology Faculty Workgroup, instituted by the GEC, remains active and the university now has 4 new courses on aging, including a geriatric Interprofessional Education (IPE) course. This forward momentum in recent years has led to the development of a new Occupational Endorsement Certificate program in gerontology. Training the future workforce and working professionals, the new program is poised to be more sustainable than a minor. In this presentation, we share details of past challenges and current successes within the College of Health.

